# Characterizing the Root Canal Configuration of Mandibular Incisors in a Western Saudi Arabian Sub-population Using Cone Beam Computed Tomography

**DOI:** 10.7759/cureus.60650

**Published:** 2024-05-20

**Authors:** Mohammed Howait, Shatha Zahran, Ayman M Abulhamael, Mohammed A Barayan, Sara Khawaji, Mohammed Sanari, Layan Alshehri, Lamees Zarei, Ammar Marghalani, Mey A Al-Habib

**Affiliations:** 1 Department of Endodontics, King Abdulaziz University, Jeddah, SAU; 2 Department of Oral and Maxillofacial Radiology, King Abdulaziz University, Jeddah, SAU; 3 Department of General Dentistry, King Abdulaziz University, Jeddah, SAU; 4 Department of Periodontology, King Abdulaziz University, Jeddah, SAU

**Keywords:** saudi sub-population, mandibular incisor, configuration, root canal, cbct

## Abstract

Aim

This study aimed to explore the morphology and complexity of mandibular anterior teeth in a Western Saudi Arabian sub-population using cone beam computed tomography (CBCT).

Methodology

CBCT scans from 818 patients were evaluated, and 3193 mandibular anterior teeth were analyzed for the number of roots, canal, canal configurations, separation level, bilateral symmetry, and gender associations.

Results

The results showed that all examined central and lateral incisors had a single root, and the majority exhibited a single canal. The prevalence of two canals in mandibular central and lateral incisors was 20.1% and 23.2%, respectively, resulting in an overall prevalence of 21.7% for two root canals in mandibular anterior teeth. The separation level of the two canals was predominantly located in the middle third of the root. Type I canal configuration was the most common, followed by type III. A high degree of bilateral symmetry in the number of canals and canal configurations was noted.

Conclusion

The findings contribute to the understanding of root canal anatomy in the Saudi population and provide valuable information for endodontic treatment planning.

## Introduction

The fundamental goals of endodontics encompass the prevention and treatment of periapical pathosis. Successful root canal therapy necessitates the identification, disinfection, shaping, and obturation of the root canal space in order to reduce microbial load [1]. To achieve these objectives, a comprehensive clinical and radiographic examination, along with accurate case diagnosis, is essential. Furthermore, a thorough understanding of the root canal morphology, complexity, and anatomical variations is crucial for optimizing treatment outcomes [2].

Numerous investigations have been conducted across diverse population groups to explore the anatomical characteristics of the lower anterior teeth. However, despite their apparent similarity, variations in canal complexity within these teeth have been observed and documented by various research groups [3-6]. Different studies have employed various methodologies to investigate root canal morphology, such as root canal staining and tooth clearing [7], conventional and digital radiography [8], and radiographic assessment enhanced with contrast medium [9]. However, advanced imaging techniques such as cone beam computed tomography (CBCT) have demonstrated superior accuracy not only in detecting periapical pathosis but also in diagnosing atypical root canal systems [10, 11].

Mandibular central and lateral incisors are generally recognized as single-rooted teeth with narrow canals [3]. However, variations can occur where dentine separates the canals within the root anatomy, either merging apically to form a single apical foramen or remaining as distinct canals with two separate apical foramina. Failure to identify the second canal, particularly the lingual canal, in root canal-treated mandibular incisors can contribute to necrosis of the missed canal and subsequent development of apical pathosis [2]. Ethnicity has been shown to contribute to anatomical variations among populations worldwide, emphasizing the need for further exploration of these variants in different regions [6, 12-14].

Numerous studies have focused on evaluating and understanding the root canal morphology and anatomy of mandibular incisors using CBCT. When evaluating 795 mandibular incisors in a Turkish population, over 50% of mandibular anterior teeth exhibited two canals, with higher incidence on the right side [15]. In a Saudi Arabian population, the evaluation of root and canal numbers, canal configurations, and gender differences in a southern subpopulation showed a collective incidence of 28.6% for two canals in mandibular central and lateral incisors, with central incisors at 26.3% and lateral incisors at 30.8% [16]. However, to date, no studies have investigated the diversity of anatomical variations in mandibular incisors within the western region of Saudi Arabia. Due to the remarkable ethnic groups' diversity within Saudi Arabia, the aim of our study is to assess the morphology and complexity of mandibular anterior teeth using CBCT in a western Saudi Arabian sub-population, utilizing different datasets.

## Materials and methods

This cross-sectional study was conducted at the Faculty of Dentistry, King Abdulaziz University, following the approval of the study protocol by the Research Ethics Committee of the same institution (No. 02-02-20). All patient's data were anonymized. 

CBCT scans previously taken for various diagnostic purposes, including impacted tooth extraction, endodontic evaluation, and oral pathology examination, were evaluated. Teeth selection criteria encompassed the absence of canal calcification, no previous root canal treatment, and no prosthetic treatments such as cemented posts or extensive coronal restorations. Scans with artifacts, blurred roots, and primary teeth were excluded.

The iCAT scanner system (Imaging Sciences International, Hatfield, Pennsylvania) was used, acquiring 8x8 cm images at 120 KVp and 5-7 mA, with a voxel size of 0.2 mm. Image analysis was performed on a calibrated Dell 17-inch high-resolution monitor with a color depth of 16-bit and a screen resolution of 1024x768 pixels. Two expert examiners, an endodontist and a radiologist, evaluated the CBCT scans using Vision software (Imaging Science International, Hatfield, Pennsylvania). In cases of disagreement, a discussion between the two examiners was conducted to reach a definitive decision.

The evaluation of mandibular central and lateral incisors involved assessing root and canal numbers, canal configuration, bilateral symmetry, and the location of canal junctions (coronal, middle, or apical). To accurately determine the level of canal split, the two expert examiners individually analyzed the CBCT images, focusing on identifying the precise point at which a single canal divided into two separate pathways. This bifurcation was categorized according to its location within the tooth: coronal, middle, or apical third of the root. The criteria for these classifications were predefined based on the relative distance from the cementoenamel junction to the apex of the root, divided equally into thirds. Discrepancies between examiners were resolved through discussion to ensure consistency and accuracy in the reporting of data. Inter-examiner reliability was assessed by randomly selecting ten CBCT images, in which root canal numbers and the type of root canal configuration were recorded. Intra-examiner reliability was measured by re-evaluating the same images after two-week intervals.

Descriptive statistics were utilized to analyze nationality, age, and gender. Intra- and inter-examiner reliability was assessed using Kappa tests. Statistical analyses, including the Pearson chi-square test and Fisher's test, were performed using SPSS software (version 22.0; IBM Inc., Armonk, New York), with a significance level set at p<0.05. The intra-consensus panel agreement and inter-examiner agreement were estimated using linear weighted Kappa's index for the number of canals and canal configurations.

## Results

The intra-examiner and inter-examiner reliability for the examiners were determined to be 0.92 and 0.88, respectively, indicating consistent and standardized evaluations and measurements performed by the two observers. The study involved 818 patients, including 516 females (63.1%) and 297 males (36.9%), with an average age of 37 ± 13.3 years and an age range of 17 to 81 years. The evaluation focused on 3193 mandibular anterior teeth, with central incisors accounting for 49.9% and lateral incisors for 50.1% of the sample.

**Figure 1:**
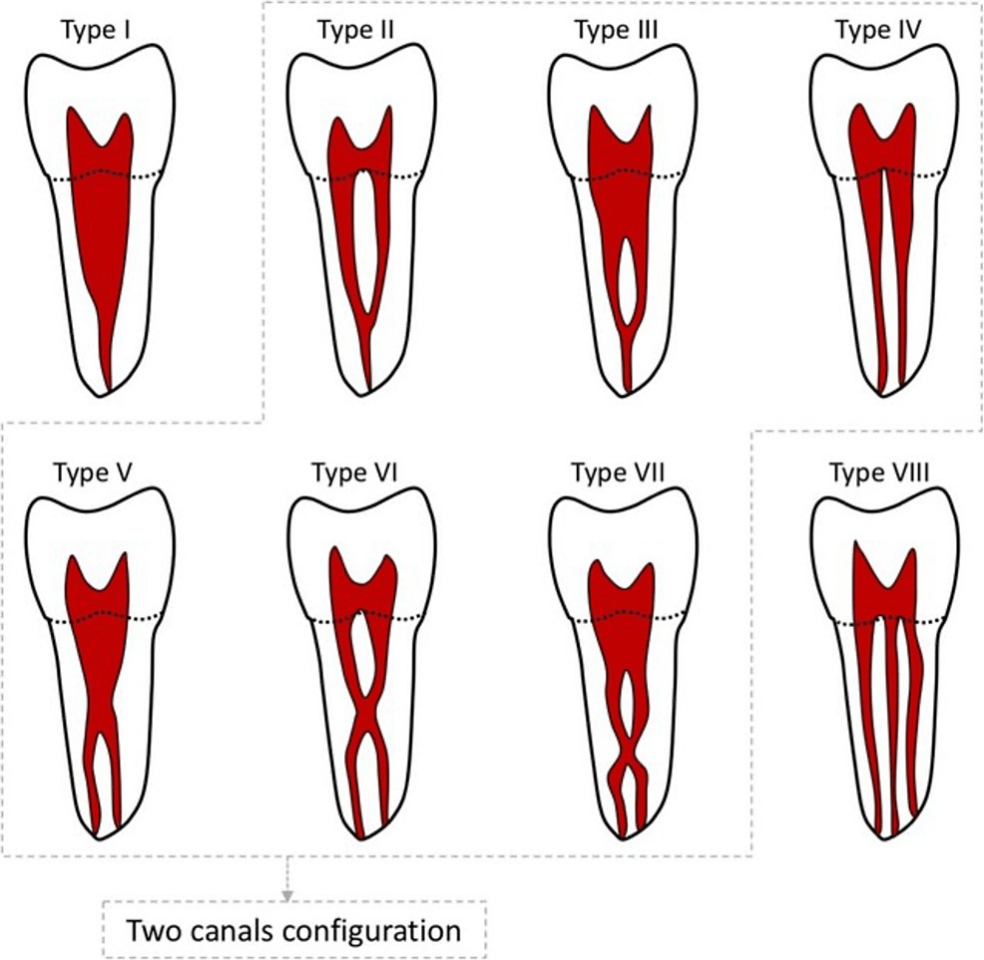
Vertucci's classification of the root canal system Source: Al Mheiri et al. [17]

All examined central and lateral incisors showed a single root. The majority of central and lateral incisors exhibited a single canal with type l Vertucci's configuration (Figure 1), representing 79.9% and 76.9% of the teeth, respectively. 

The prevalence of two root canals in mandibular central and lateral incisors was found to be 20.1% and 23.2%, respectively, resulting in an overall prevalence of 21.7% for two root canals in mandibular anterior teeth. In teeth with two canals, the separation site was almost exclusively located in the middle third (99%), to a much lesser extent in the coronal and apical third (0.6% and 0.4%, respectively). Further details on the number of canals and canal morphology are shown in Table 1.

**Table 1:** Descriptive statistics of examined central and lateral incisors (n=3193 anterior teeth), presented as number of teeth (%) Canal configuration based on Vertucci's classification.

	Central incisors	Lateral incisors	All teeth
Number of canals			
One canal	1272 (79.9)	1229 (76.8)	2501 (78.3)
Two canals	320 (20.1)	372 (23.2)	692 (21.7)
Canal configuration			
Type I	1272 (79.9)	1231 (76.9)	2503 (78.4)
Type III	316 (19.8)	368 (23)	684 (21.4)
Type V	2 (0.1)	2 (0.1)	4 (0.1)
Type II	2 (0.1)	1231 (76.9)	2 (0.1)
Level of split (teeth with two canals only n=692)			
Apical	2 (0.6)	1 (0.3)	3 (0.4)
Middle	316 (98.8)	369 (99.2)	685 (99)
Coronal	2 (0.6)	2 (0.5)	4 (0.6)
Total	1593 (49.9)	1600 (50.1)	3193

Overall, type I canal configuration was the most common, followed by type III, while type II and V configurations were observed to a much lesser extent, as shown in Table 1.

Among the 818 patients included in the study, when examining the right and left mandibular central and lateral incisors, 96.3% of the teeth exhibited symmetrical numbers of canals and canal configurations.

## Discussion

Root canal treatment success relies on a comprehensive clinical and radiographic examination, precise diagnosis, and understanding of root canal anatomical complexities. These complexities encompass bifurcations, accessory canals, and apical deltas [18]. Thus, the objective of this study was to explore the morphology of mandibular anterior teeth and the number of canals in the Saudi population utilizing CBCT. The outcomes of this investigation contribute to the existing knowledge of dental anatomy by shedding light on root and canal numbers, canal configurations, separation levels, bilateral symmetry, and gender associations. The intention is to enhance the effectiveness of endodontic treatment.

Analysis of tooth morphology in the Saudi population revealed that all examined central and lateral incisors had a single root, consistent with prior studies in various populations [5, 6, 11, 12, 19, 20]. Vertucci's seminal work on 300 mandibular incisors, utilizing a clearing technique with dye injection, reported a prevalence of two canals in 30% and 25% of mandibular central and lateral incisors, respectively [3]. Similarly, in the present study employing CBCT, the majority of central and lateral incisors exhibited a single canal, accounting for 78.3%. The prevalence of two canals was observed in 21.7% of the sample, with a slightly higher tendency in lateral incisors compared to central incisors. When compared to global numbers, although numerous studies revealed comparable figures [5, 6, 12, 19-21], other geographical areas reported a higher incidence of second canals [5, 15, 22]. For example, a study on a Turkish population revealed that over 50% of 795 mandibular incisors had two canals, predominantly on the right side [15]. Other geographical areas reported as low as 3.8% prevalence [14].

In regard to the Saudi population, investigations conducted on mandibular incisors from both northern and southern subpopulations, utilizing the decalcification technique and CBCT imaging, demonstrated a slightly higher prevalence of two canals compared to the current study, approximately 30% [16, 23]. 

Regarding the site of canal separation in mandibular anterior teeth, the middle third of the root was predominantly identified as the location (99%), while occurrences in the apical and coronal regions were less frequent. This finding is somehow consistent with existing literature [5, 22] emphasizing the importance of understanding canal separation sites for successful root canal treatment planning.

**Figure 2:**
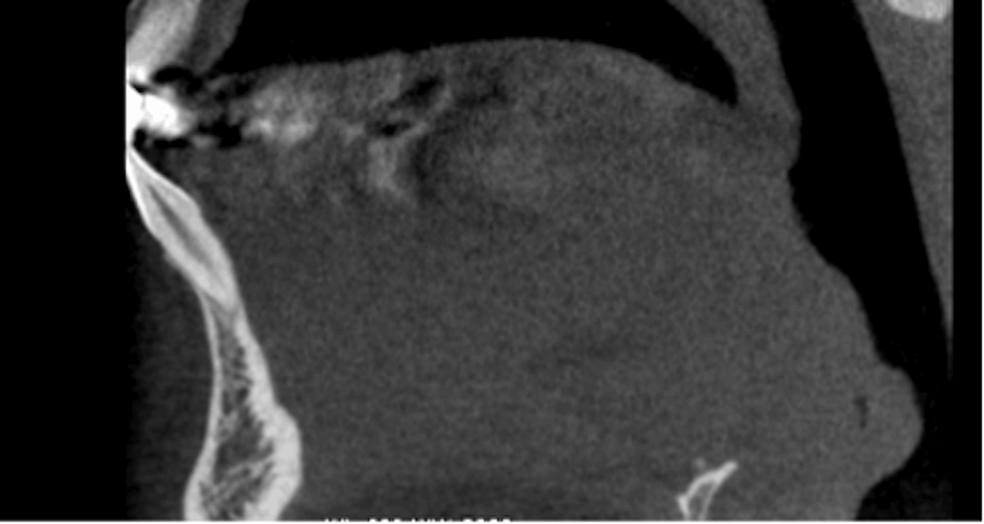
Sagittal view of vertucci classification type I of the mandibular incisor

**Figure 3:**
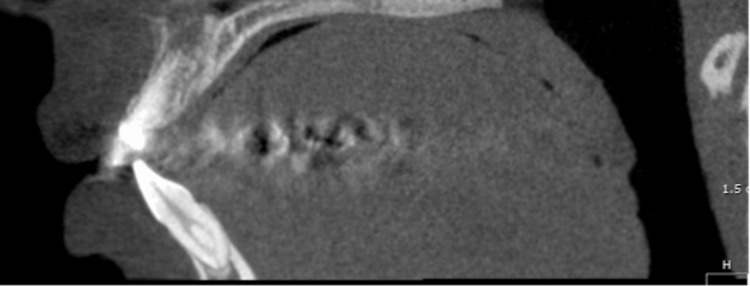
Sagittal view of type III Vertucci's classification of the mandibular incisor

Canal configurations were analyzed, with type I (single canal; Figure 2) being the most prevalent in both central and lateral incisors, which is in line with previous studies [5, 16, 22-24]. Type III (Figure 3) configuration represented the second most prevalent, accounting for approximately 21% of anterior teeth, while type II and V configurations were rarely encountered. These findings are valuable for clinicians in endodontic treatment planning, underscoring the significance of considering anatomical variations in canal configuration. 

Bilateral symmetry in the number of canals and canal configurations between the right and left mandibular anterior teeth was assessed and found to be present in the majority of cases (96.3%). This finding aligns with established studies [5, 13, 15], indicating the bilateral symmetry of mandibular anterior teeth and supporting the notion that information obtained from one side can be extrapolated to predict the anatomy of the contralateral side.

To enhance the comprehension of root canal morphology, CBCT scanning was employed in this study. CBCT provides highly accurate 3D images with minimal distortion, facilitating the identification of all canals and minimally invasive access cavity preparation [25]. However, CBCT has limitations, such as potentially compromised image quality due to factors like patient movement, restorations, and radiopaque materials, leading to scatter and beam hardening artifacts that may affect diagnostic accuracy [26].

The limitations of this study include data collection from a single centre. In future investigations, collaboration among multiple centers and a larger sample size would yield more precise insights into root canal anatomy within the Saudi population. Additionally, the study focused exclusively on the Saudi population in the western region, cautioning against generalizing the findings to other ethnicities within the country, given the wide range of ethnic backgrounds in this region. Moreover, employing limited view CBCT with improved resolution and voxel size would enhance image quality and aid in the observation of root canal morphology, particularly in mandibular anterior teeth.

## Conclusions

In conclusion, this study provides valuable insights into the morphology of mandibular anterior teeth and the number of canals in the Saudi population using CBCT imaging. The findings align with previous studies, indicating the predominance of type I canal configuration and the presence of a second canal in a significant proportion of central and lateral incisors. The knowledge gained from this study can assist clinicians in making informed decisions and optimizing endodontic treatment outcomes in the Saudi population. While CBCT imaging proves valuable in enhancing our understanding of root canal morphology, its limitations should be acknowledged. Further research with larger sample sizes and diverse populations is necessary to advance our comprehension of tooth anatomy and canal variations.
